# A Perinatal Mental Health Education Programme for Maternity, Neonatal and Other Allied Multidisciplinary Teams in NHS Lothian: Why, How, and What's Next

**DOI:** 10.1192/bjo.2022.104

**Published:** 2022-06-20

**Authors:** Vikki Argent, Megan Sherratt, Marian Nelson

**Affiliations:** NHS Lothian, Edinburgh, United Kingdom

## Abstract

**Aims:**

Throughout the development of the NHS Lothian Perinatal Mental Health Service, their goals alongside maternity and neonatal teams, family nurse and health visiting services have been to strengthen interdisciplinary working and improve the quality of perinatal mental health care delivered to birthing people. The aims of developing a programme of multidisciplinary education sessions were to develop the knowledge and confidence of non-mental health professionals in caring for birthing people experiencing mental health difficulties, and aid understanding of available services and referral pathways to facilitate appropriate care.

**Methods:**

The programme has been delivered by a Perinatal Psychiatrist and Clinical Midwifery Educators in bimonthly sessions lasting two hours. Sessions have included scenario-based learning, education regarding illnesses and disorders, and promotion of infant mental health and trauma-informed care.

Participants have attended virtually via Microsoft Teams or in person. Sessions have been recorded and accessed following teaching. Confidentiality is upheld throughout. A standard operating procedure utilising multi-modal methods has been designed to maximise staff engagement with sessions. Feedback accessed via a QR code has been collated via a Microsoft Forms questionnaire comprising of Likert scale and free-text answer questions. Feedback has guided programme development and topic selection.

Initially, sessions were open to inpatient maternity services, but now outpatient maternity services, neonatology, and health visiting and family nursing colleagues are invited to maximise the reach of the staff delivering sessions.

**Results:**

Sessions have been well accessed by a variety of professionals, with increasing attendance at each session. Feedback has been obtained from 43% of those attending. Results have been very positive: 100% of respondents strongly agreed or agreed that the format of the session worked well with 92% of respondents strongly agreeing or agreeing that the session content was pitched at the appropriate level. 100% of respondents felt that the content covered was useful in their clinical role which supports the emphasis of the sessions on linking knowledge to clinical application to build confidence. 100% of respondents would recommend these education sessions to a colleague.

**Conclusion:**

Feedback to date has shown that participants have found the sessions to be accessible and the content appropriately pitched and clinically valuable. Despite launching the programme amidst the challenges of COVID-19, participants have found the delivery of the sessions to be supportive and collaborative.

The next phase in the development of the programme will be to understand in more detail what participants are learning and the impact on their practice.

